# *In silico* re-assessment of a diagnostic RT-qPCR assay for universal detection of Influenza A viruses

**DOI:** 10.1038/s41598-018-37869-w

**Published:** 2019-02-07

**Authors:** Alexander Nagy, Tomáš Jiřinec, Helena Jiřincová, Lenka Černíková, Martina Havlíčková

**Affiliations:** 1 State Veterinary Institute Prague, Prague, Czech Republic; 2 National Institute of Public Health, National Reference Laboratory for Influenza, Prague, Czech Republic

## Abstract

The ongoing evolution of microbial pathogens represents a significant issue in diagnostic PCR/qPCR. Many assays are burdened with false negativity due to mispriming and/or probe-binding failures. Therefore, PCR/qPCR assays used in the laboratory should be periodically re-assessed *in silico* on public sequences to evaluate the ability to detect actually circulating strains and to infer potentially escaping variants. In the work presented we re-assessed a RT-qPCR assay for the universal detection of influenza A (IA) viruses currently recommended by the European Union Reference Laboratory for Avian Influenza. To this end, the primers and probe sequences were challenged against more than 99,000 M-segment sequences in five data pools. To streamline this process, we developed a simple algorithm called the SequenceTracer designed for alignment stratification, compression, and personal sequence subset selection and also demonstrated its utility. The re-assessment confirmed the high inclusivity of the assay for the detection of avian, swine and human pandemic H1N1 IA viruses. On the other hand, the analysis identified human H3N2 strains with a critical probe-interfering mutation circulating since 2010, albeit with a significantly fluctuating proportion. Minor variations located in the forward and reverse primers identified in the avian and swine data were also considered.

## Introduction

Quantitative polymerase chain reactions and reverse transcription polymerase chain reactions (qPCR/RT-qPCR) utilizing 5′ nuclease or hydrolysis probes^1^, also known as TaqMan qPCR/RT-qPCR, represent the primary screening tools in diagnostic microbiology. A fully optimized and validated diagnostic qPCR/RT-qPCR assay should sensitively and specifically detect the selected nucleic acid target with high amplification efficiency. It should also be sufficiently inclusive, i.e., to enable the detection of as many genetic variants, subtypes, strains and phylogenetic lineages of a given microbial pathogen as possible. This property is, however, greatly influenced by extensive genetic variability, mainly of the viral genomes^2–12^. The inclusivity of a diagnostic qPCR/RT-qPCR assay is, therefore, an important quality parameter which should be periodically re-assessed. Also, the adoption of a previously developed qPCR/RT-qPCR assay in the laboratory should optionally be accompanied by *in silico* primers and a probe re-assessment on the public sequences to evaluate the ability to detect actually circulating strains and to infer the potentially escaping variants.

In 2010, we developed and evaluated an RT-qPCR assay intended for the universal detection of IA viruses from avian and mammal species^13^. Based on a large-scale multiple sequence alignment (LS-MSA), including more than 10,000 M-segment sequences from all available host species, a pair of highly conserved primers and a short locked nucleic acid-modified probe were selected. Since the assay became one of the recommended methods for the generic detection of the Avian Influenza virus by the European Union Reference Laboratory for Avian Influenza and around ten times more data have been accumulated since the original design, we found it important to re-evaluate the primer and probe sequences *in silico* on a currently available collection of IA virus M sequences.

A LS-MSA represents a suitable platform for the *in silico* identification of long-term trends in nucleotide variability in the selected portions of the genome of interest. Therefore, a comprehensive LS-MSA should be the first step in oligonucleotide selection during the diagnostic qPCR/RT-qPCR assay design. Conversely, a previously developed assay can be challenged against the contemporary sequences to infer the actual “power” *in silico* before its implementation in routine use.

To provide assistance to microbiologists with diagnostic qPCR/RT-qPCR assay design, we provided a quick and simple pipeline for LS-MSA evaluation and the identification of the evolutionarily conserved positions^14^. The procedure was set up from the perspective of a diagnostic laboratory and requires no extensive bioinformatical or computational skills. It can be performed on an ordinary internet-connected desktop computer, and it was also used in the study presented. However, the *in silico* re-assessment required additional descendant tools enabling quick sequence sorting and filtering through the LS-MSA. For instance, if an LS-MSA, counting thousands of lines, reveals an interesting variation at potentially critical primer or probe positions, it is laborious to sort out the corresponding sequences from the huge body of the LS-MSA for a detailed study. When only a few such sequences are dispersed throughout the entire LS-MSA, this process is even more difficult and can be likened to searching for a needle in a haystack. To simplify and accelerate this process in the presented methodology report we also introduce a web-based tool, called the SequenceTracer, designed for simple alignment stratification, compression and sequence sorting.

From this perspective our aims were to:

(i) Download and align the entire collection of IA virus M sequences from all host species available,
(ii) Evaluate nucleotide variability within the primers and probe regions,
(iii) Identify the number and frequency of primer-probe-primer sequence variants across the data,
(iv) Re-assess assay inclusivity for the main host categories *in silico*,
(v) Identify and filter out the IA virus strains that could potentially decrease assay fitness or entirely escape detection and provide their spatial and temporal investigation,
(vi) Demonstrate the utility of the SequenceTracer in the *in silico* re-evaluation of the primer and probe sequences.

## Results

### Background information

The IA virus is an enveloped virus with a genome comprised of eight negative sense RNA segments. Taxonomically, the IA virus belongs to the family *Orthomyxoviridae* and is further divided into subtypes according to the combination of the two main surface antigens, hemagglutinin (HA) and neuraminidase (NA). Currently, 16 standard HA subtypes (H1–H16) and 9 standard NA subtypes (N1–N9) are known to potentially allow 144 various combinations. The IA-like viruses of the H17N10 and H18N11 subtypes identified in bats^15,16^ were not included in the presented study.

RT-PCR-based assays for the universal detection of the IA virus are conventionally targeted on the M-segment regions, which are highly conserved across various subtypes and host species. The M-segment is a 1,027 nucleotide-long bicistronic molecule which encodes two membrane proteins, M1 and M2. The long-term evolution of the entire segment is characterized by the continuous accumulation of nucleotide mutations, without insertion or deletion mutagenesis^17,18^. Based on the evolutionarily highly conserved regions of the M-segment, a pair of 17 nucleotide-long primers, encompassing the amplicon of 182 nucleotides in length, and an eight nucleotide-long TaqMan probe were selected^13^. The short probe motif was found to be extremely conserved. Nevertheless, it did not tolerate any mutations in the corresponding template region. Mutations in this region are fatal with definite false negativity^13,19^. We investigate the persistent ability of the RT-qPCR assay to universally detect the IA viruses.

### Definition of the data set

The SVIP-MP RT-qPCR assay was re-assessed *in silico* on a comprehensive dataset of 99,353 IA virus M-segment database submissions divided into five datasets: Human H3N2, Human pandemic H1N1 (H1N1pdm), Avian, Swine, and Others (see Table 1).

**Table 1:** The sequences included in the study.

		Source	Downloaded	Considered vs. total number of groups	Informative	Final	Inclusivity
**Human H3N2**		GISAID	37,406	2/35	36,086 (97.5%)	35,938 (99.6%)	32,944 (91.7%)
**Human H1N1pdm**		GISAID	16,948	1/30	16,484 (97.3%)	16,344 (99.2%)	16,344 (100%)
**Avian**		GISAID	21,530	4/100	21,063 (97.8%)	20,750 (98.5%)	20,541 (99%)
**Swine**		IVD	9,337	5/54	9,290 (99.5%)	9,139 (98.4%)	8,633 (94.5%)
**Others**	Others subtotal		7,064	2/34	6,031 (85.4%)	5,958 (98.8%)	5,958 (100%)
	Human H1N1 seasonal	IVD	3,140				
	Human H7Nx	GISAID	1,249				
	Human H5Nx	GISAID	428				
	Human H2N2	IVD	129				
	Human H1N2	GISAID	71				
	Human H9N2	GISAID	22				
	Human H10N8	IVD	6				
	Environment	GISAID	1,368				
	Canine	IVD	303				
	Equine	GISAID	238				
	Feline	GISAID	44				
	Other mammals	GISAID	70				
**TOTAL**			**99**,**353**	**—**	**88**,**957 (89**.**5%)**	**88**,**129 (99**.**1%)**	**84**,**420 (95**.**8%)**

The Human H3N2 data included 37,406 sequences and reflected the entire known history of the “Hong Kong flu” in the human population from 1968 to February 2018.

The Human H1N1pdm pool contained 16,948 submissions, i.e. the entire M-segment data of pandemic influenza collected throughout the world since its first emergence in 2009 up to February 2018.

The Avian set was comprised of 21,530 sequences which encompassed all known avian IA virus subtypes collected from a plethora of wild and domestic bird species from different geographic areas and from both hemispheres from 1902 to February 2018. It also contained sequences from the low and highly pathogenic avian influenza virus outbreaks reported during this period worldwide, including the recent 2014–2017 global, highly pathogenic H5N8 and 2018 H5N6 strains.

The Swine IA virus M-segment data pool consisted of 9,337 sequences collected from 1930 and contained all known IA virus subtypes detected in swine so far. Beside the enzootic H1N1, H1N2, and H3N2 subtypes, including the classical swine H1N1, avian-like swine H1N1, double, and triple H3N2 reassortants, the swine IA M data set also contained avian-like IA virus sequences like H2N3, H4N1, H4N6, H4N8, H5N1, H5N2, H5N6, H6N6, H7N2, H9N2 and H11N6 subtypes resulted from opportunistic transmissions from the avian reservoir to swine.

The Others set encompassed 7,064 M sequences from various IA virus subtypes and sources, including 129 human H2N2 collected between 1957 and 1968 (Asian flu), 3,140 human M sequences from 1918–2016 representing the H1N1 “Spanish flu” and “Russian flu” pandemics, and 71 human H1N2 that circulated during 1967–2016. Next, zoonotic IA viruses, such as 428 human H5Nx, 1,249 H7Nx, 22 H9N2, and 6 H10N8 M sequences, were included. Finally, the Others collection was supplemented with 1,368 environmental, 303 canine, 238 equine and 44 feline sequences and also with 70 submissions from minority species like seal, ferret, stone marten, mink, pika, muskrat, camel, panda, yak and raccoon.

The heterogeneity of the downloaded data sets was further enhanced by the IA virus sequence origin from a plethora of various sources, including clinical specimens such as blood, various organs, faeces, bronchoalveolar lavages, various swabs (cloacal, nasopharyngeal, buccal, surface) through environmental specimens (faeces, water) as well as *in vitro* culture matrices like embryonated chicken eggs, different cell lines, and experimentally infected animals.

### Evaluation of nucleotide variability in primers and probe-binding regions

**Figure 1:**
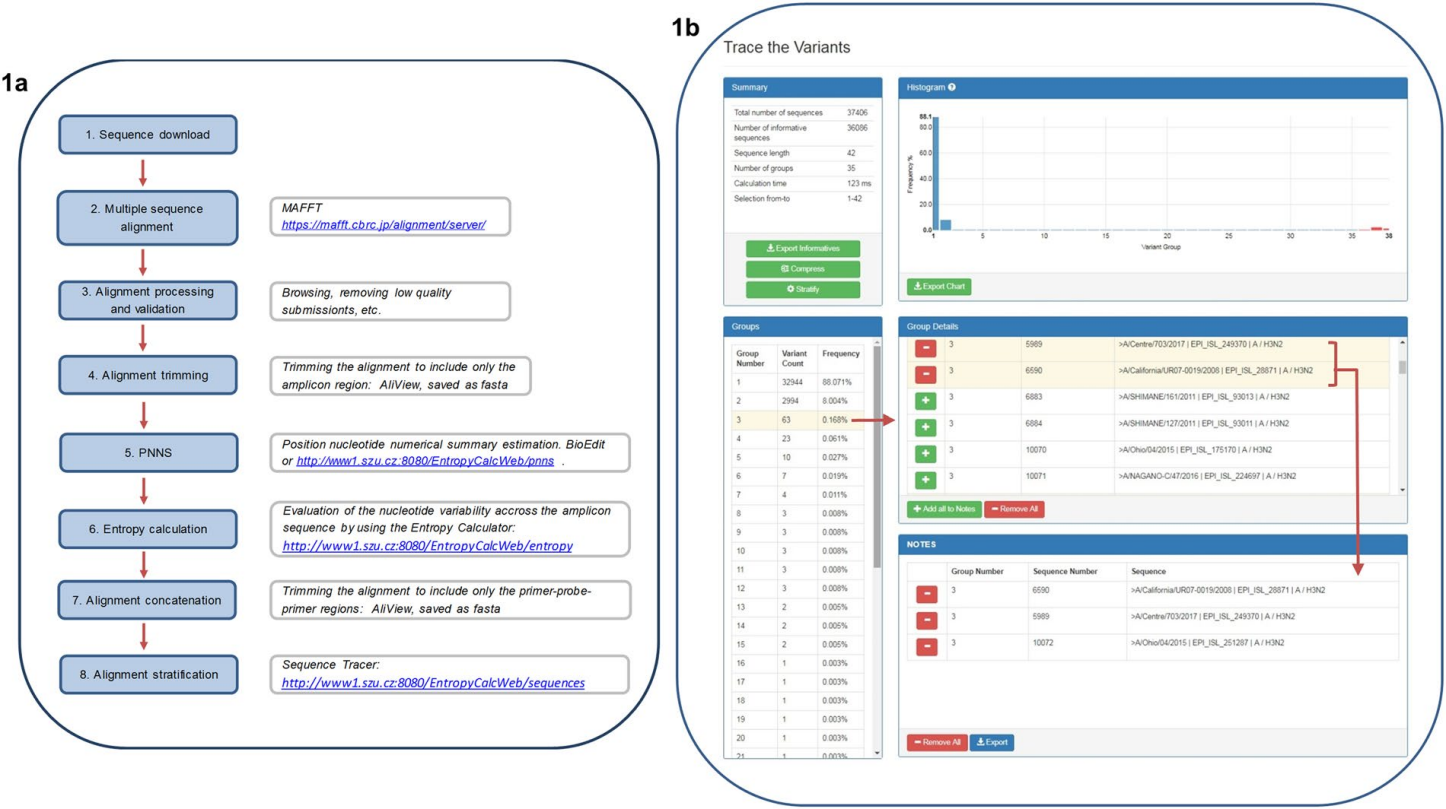
The sequence tracing pipeline. The (**a**) illustrates the sequence tracing pipeline from sequence download to alignment stratification used in the study. The (**b**) shows the alignment stratification procedure on the concatenated Human H3N2 M-segment pool as an example uploaded to the SequenceTracer as a FASTA file. A list of sequence groups in descending order along with the number of strains/group and their percentage frequencies are summarised in the “Groups” column. For clarity, the frequency of each group is visualised in a column chart. By selecting a specific group (no. 3 in the figure), its content appears in the “Group Details” window as a list of FASTA header lines along with the group number and the sequence number in the alignment. The sequences of interest, or the entire group contents, can be copied to the “Notes” and exported as a FASTA file, thus creating the user’s own data set.

First of all, the particular datasets were aligned, and the resulting LS-MSAs were processed and validated (Fig. 1a; steps 1–3). Next, the alignments were trimmed to include only the SVIP-MP assay 182 bp-long amplicon (Fig. 1a; step 4). Finally, sequence variability in the entire amplicon was investigated across each of the five datasets by calculating the positional nucleotide numerical summary (PNNS) and entropy (Fig. 1a; steps 5 and 6).

**Figure 2:**
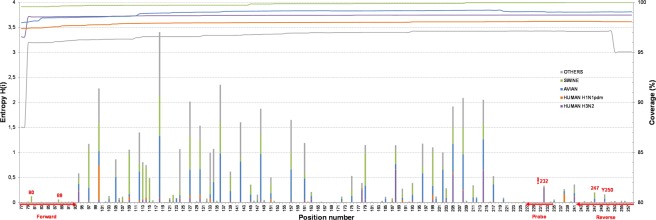
The entropy plot. The nucleotide variation in each position of the 182 bp RT-qPCR amplicon (M-segment positions 77–258), calculated as entropy, was visualised separately for the five data pools: Human H3N2 (purple), Human H1N1pdm (red), Avian (blue), Swine (green), and Others (grey) as a column chart related to the primary y-axis. The particular column heights are proportional to the amount of nucleotide variation per data pool. The primers and probe-binding positions, along with the positions of interest, have been highlighted in red. The line chart designates the positional coverage of the 182 bp-long RT-qPCR amplicon is related to the secondary y-axis.

All five data sets exhibited high density, since almost the entire amplicon was covered by more than 95% of the sequences (Fig. 2), i. e., at least 95% of the data in each particular LS-MSA contained both the primer and probe motives. The only difference was observed at the first two positions of the Others pool, which was slightly less covered (87.5%).

The entropy plot (Fig. 2), summarising the amount of sequence variation in each position, suggested that the primers and probe-binding regions retained extremely high sequence stability across the entire dataset.

The entropy plot further revealed a slightly increased variation in five positions: (i) forward primer positions of the Swine M sequence pool with C80T and A88G transitions; (ii) probe position 232 of the Human H3N2 data with the C232A transversion; and (iii) reverse primer position 247 of the Swine and Avian M sequence pools with G247A, T and G247A changes. A detailed position variability profile for each data pool is provided in Supplementary Information, Fig. S1.

### Alignment stratification and sequence tracing

The most significant finding of the entropy plot is, however, the increased variation in probe position 232 observed within the Human H3N2 pool. Although the percentage proportion of these C232A M-segment variants is relatively small, 8%, they are a source of concerns due to their interference with probe binding and false negativity^13,19^. Hence, it is important to investigate in more detail which IA virus strains held the C232A mutation. So, what does the 232A Human H3N2 M-segment population look like? Is it randomly distributed or structured? Is the C232A transversion dispersed randomly throughout the data, or is it related to certain epidemic seasons or geographic areas? In addition, what are the patterns of the mutations observed in the primer regions?

To sort out the particular strains of interest, or more generally, to stratify the LS-MSA in order to reveal the total number of sequence variants, we developed a web-based application called the SequenceTracer (Fig. 1b). The SequenceTracer divides the LS-MSA, or the selected portions of it, into discrete groups of identical sequence variants. Then, the sequence count in each group and the group frequency percentage are calculated. The groups are numbered and listed in descending order. In parallel with sequence stratification, a graphical output in the form of a column diagram, which represents the frequencies of the particular variants as a function of the group number, is prepared. As a result, the SequenceTracer provides a quick overview of the sequence variant composition of the LS-MSA.

Once the LS-MSA is deconvoluted into its constituent elements, the SequenceTracer enables group browsing and sequence sorting options. Each variant group can be further explored for specific IA virus strain content. Next, the particular sequences of interest or entire groups can be copied to the “Notes”, thus creating the user’s own personal subset. Finally, the content of the Notes can be exported as a FASTA file and browsed with the user’s preferred alignment editing software. The whole process of sequence tracing is fully intuitive, and the results are clearly interpretable without the necessity of extensive computational or biostatistical skills. Additional features are represented by the following functions:

- Compress - prepares a weighted alignment by selecting a representative sequence of each variant group. Then, for each group, a weight (frequency) is assigned which reflects the abundance in the original LS-MSA.
- Stratify - calculates the number and frequency of each group with a graphical output.
- Export informatives - exports all informative sequences, i.e. all sequence groups except outgroups 1, 2, and the “excluded” submissions.

### *In-silico* re-assessment of the SVIP-MP RT-qPCR assay

To explore the IA virus strains holding the particular nucleotide changes within the primers and probe sequences, the SVIP-MP amplicon containing LS-MSAs, were further cut to include only the forward primer-probe-reverse primer motifs (further referred as region of interest (ROI); Fig. 1, step 7). Such, concatenated, LS-MSAs of all five IA virus M sequence sets were stratified by using the SequenceTracer (Fig. 1b).

**Figure 3:**
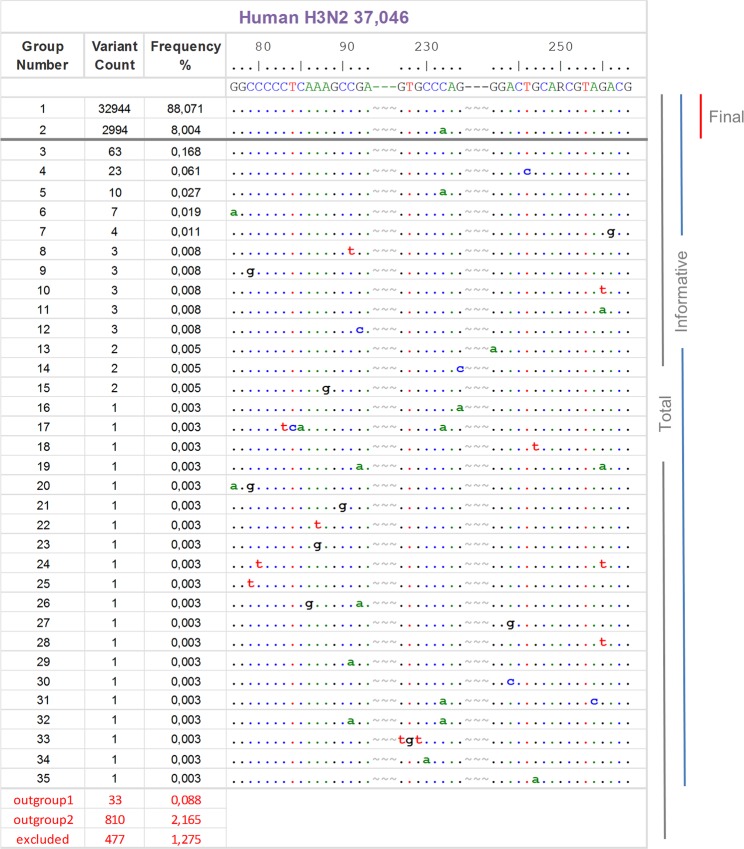
Alignment stratification of the concatenated Human H3N2 M data pool. The 37,406 concatenated (forward-probe-reverse) sequences were decomposed into individual sequence variants by using the “Stratify” function of the SequenceTracer. The figure shows the number of sequence variants and the frequency of each variant in a descending order along with a representative sequence of each group aligned to the primers and probe-binding regions (5′ → 3′). Note that the original probe is in reverse direction, i.e. CTGGGCAC. The alignment was prepared in the Graphic View mode of the BioEdit program. For clarity, the primers and probe sequences were separated by tildes and numbered according to their M-segment positions. The dots indicate an identical nucleotide. The horizontal grey bar designates the threshold (≥0.5%), and the vertical bars highlight the total, informative and final sequence groups. “Outgroup 1” contains sequences with at least one uncertainty (R, Y, N, etc.), and “outgroup 2” encompasses incomplete submissions. The “excluded” submissions did not include the regions of interest. For alignment stratification of the remaining IA virus M sequence pools, please refer to Supplementary information, Fig. S3.

The analysis revealed that the concatenated H3N2 LS-MSA contained 35, the H1N1pdm 30, the Avian 100, the Swine 54, and the Others pool 34 different ROI groups (Fig. 3, Table 1; Supplementary Information, Fig. S2a,d). Further, as was indicated by the PNNS and entropy plot, the alignment stratification exhibited a highly unequal distribution of the data, where 84–96% of the ROIs, in dependence of the data pool, were identical to the primers and probe sequences. Then, the frequencies of the deviating ROIs sharply decreased where numerous variants were represented by one or a few submissions. The sharp decrease in frequency enabled setting a threshold where only the primer-probe-primer motifs with ≥0.5% incidence were further considered.

The threshold definition further enabled defining assay inclusivity separately for each of the five data sets and also to establish the overall inclusivity. To this end, the “Informative” ROIs were counted first: the total sequence number per M-segment pool was subtracted by “outgroup 1” (submissions containing ambiguous positions), “outgroup 2” (short stretches of the ROI), and “excluded” (submissions which did not include the ROI). Next, the “Final” set was defined as those from the “Informative groups”, situated above the threshold. Finally, inclusivity was determined as the proportion of the Final ROI group with 100% identity to the primers and the probe (Fig. 3). As a result, the *in silico* analysis showed that the re-assessed assay is apparently capable of detecting at least 91.7% of known human H3N2, 100% of human H1N1pdm, 99% of Avian, 94.5% of Swine and 100% of Others M sequences. Overall, this means at least a 95.8% detection rate at the defined threshold level (Table 1).

### Spatiotemporal investigation of the IA virus strains with mutations in the primers and probe regions

**Figure 4:**
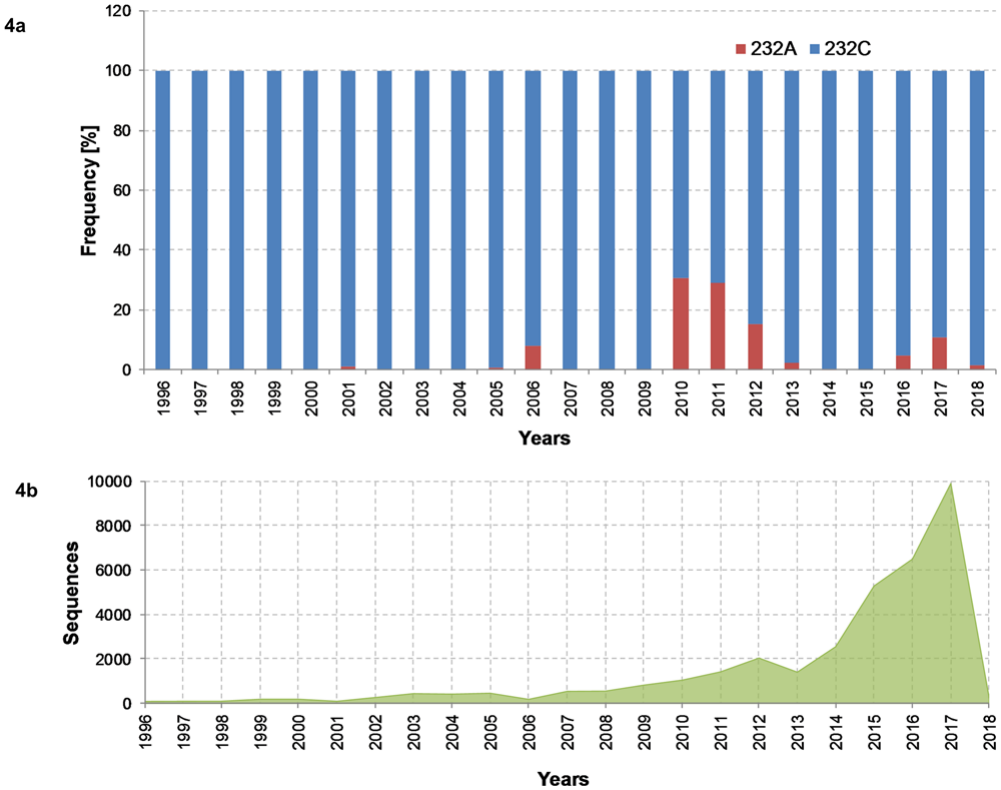
Temporal distribution of the C252A Human H3N2 M-segment variant. The column chart (**a**) shows the temporal distribution of the C232A Human H3N2 M-segment variant (Fig. 3 group 2) and its percentage proportion among the submitted sequences in the GISAID database per given year. In the (**b**), the total number of submitted sequences per year from 1996 to February 2018 is given in absolute numbers.

The Human H3N2 LS-MSA stratification showed that the C232A variants were observed in ROI groups 2 and 5 containing in summary 3,004 sequences (Fig. 3). The SequenceTracer was used to extract the submissions belonging to these groups, and the spatial and temporal characteristics were investigated. The analysis (Fig. 4) revealed that the C232A Human H3N2 strains have been appearing in the data since 2001 with a slight peak in 2006. However, the proportion of these strains increased substantially during the 2010–2011 influenza seasons, where they constituted 30.7% (463/1,506) and 29.1% (582/1,998) of the submissions of apparently worldwide occurrence. Then, the proportion of the C232A mutants started to decrease to 15.5% (373/2,036) in 2012 and 2.4% (35/1,437) in 2013. In 2014, only three H3N2 strains out of 2,548 submissions (0.1%) held the C232A mutation, and in 2015 its appearance had not been detected among 5,277 submissions. Nevertheless, the C232A variants were evidently persistent in the global Human H3N2 population, as was suggested by their redetection in 2016 with the frequency of 4.8% (324/6,810) which increased in 2017 to 10.8% (1198/11,080). C232A H3N2 strains were also observed in 2018 with 1.7% frequency (4/239; 27 February 2018).

The alignment stratification of the remaining M data pools also enabled a more detailed view on the nucleotide variations in the ROIs mentioned above. Namely, at the defined threshold, we observed two M-segment ROIs bearing changes within the forward primer (Swine-group 2 C80T and group 5 A88G; Supplementary Information 1, Fig. S2b) and three within the reverse primer-binding positions (Swine-group 3/Avian-group 4 G247T, and Avian-group 3 G247A; Supplementary information 1, Fig. S2b,c). Especially, the forward primer A88G and the reverse primer G247T/A mutations are of concern since they may impair 3′ end priming and decrease the assay sensitivity. Sequence tracing showed that these M-segment variants were limited only in certain localities or outbreak areas.

Within the Swine M data, the A88G and G247T ROI variants occurred with a frequency of 2.6% and 0.7%, respectively, almost exclusively in the North American swine of various subtypes circulating between 2011 and 2016. Similarly, the Avian G247A variant (group 3), observed with a frequency of 0.3%, dominated in North America mainly in H4N6, H10N7 and H3N8 subtypes from interior Alaska in 2008–2009. In a minority, this variant was also observed among different subtypes from New Jersey, Delaware and California. On the other hand, the Avian G247T variants, with a frequency of 0.5%, were comprised mainly from Korean H9N2 viruses detected in poultry between 2003–2009.

## Discussion

The ongoing evolution of microbial pathogens, accompanied by the continuous accumulation of nucleic acid changes, represents a significant issue in diagnostic PCR/qPCR. A comprehensive review^2^ revealed that many assays developed before 2008 are burdened with high false negative rates, due to mispriming and/or probe-binding failures. Interestingly, a significant fraction of the PCR/qPCR assays listed in the World Organisation for Animal Health manuals^20^ have been dated from that same period. The poor inclusivity of the pre-2008 assays was particularly inevitable, due to the lesser volume of sequence data available at that time for oligonucleotide selection. On the other hand, it also resulted from poor primers and probe design.

However, the primer and probe mismatches greatly influence the fitness of the relatively recent assays as well^2–12^. In addition, the authors often do not re-evaluate their previously published PCR methods. These arguments are strongly indicative of the necessity of the *in silico* oligonucleotide re-assessment of diagnostic PCR/qPCR assays before their implementation in routine use. One option to fulfil this criterion is to study the publicly available sequence data using LS-MSA.

Here we proposed a procedure on how to use the LS-MSAs for the assay re-evaluation *in silico* and demonstrated it on an RT-qPCR assay for the detection of IA viruses^13^. We analysed more than 88,900 informative IA virus M-segment sequences divided into five different data pools. The data represented the entire information content of the GISAID and IVD databases as of 27 February 2018.

Generally, the investigation of sequence stability within the primers and probe-binding regions revealed excellent inclusivity for the majority of the datasets at the defined threshold level. These inclusivity rates are comparable with the previous data, obtained ten years ago and on a ten times smaller dataset^13^, and confirm the high evolutionary stability of the proposed oligonucleotide sequences across different IA virus strains, subtypes, pathotypes and host species. The results also underline the essential importance of a comprehensive *in silico* primer and probe design strategy in diagnostic PCR/qPCR assay design.

Despite the high inclusivity rates, we observed a marked decrease in the human H3N2 data pool, from the original 99.5% to 91.7%, due to the critical, probe-interfering mutation C232A. The H3N2 strains with this change escape detection^13,19^. An analysis of the temporal distribution showed that the C232A H3N2 M variants had also been presented in the original dataset, albeit at a very subtle background level. Paradoxically, by an irony of fate, their proportion started to significantly increase among the human H3N2 strains immediately after the assay had been released. The temporal analysis also suggested apparent fluctuations of the C232A H3N2 strains between epidemic seasons, at least it could be inferred from the submitted data. Since the C232A variants constituted ~11% of the human H3N2 M-segment submissions for the 2017 epidemic season and may represent a considerable fraction of the actually circulating human H3N2 strains as well, we have concluded that the current version of the re-assessed RT-qPCR assay is insufficient for being used as a method for surveillance of the H3N2 subtype in the human population. However, given the still high detection rate of the assay, 91.7%, it would be interesting to compare its inclusivity relative to other currently used methods for the generic detection of seasonal IA viruses in humans.

Besides the most striking human H3N2 instance, a slight decrease in the *in silico* inclusivity in the Swine data pool from the original 98.9% to 94.5% was also observed. The sequence tracing suggested that this decrease was caused by minor oscillations mainly in reverse primer position G247T and, in particular, in forward primer position A88G. Both of them were inferred as critical, since they were positioned towards the 3′ ends and may compromise elongation. Spatiotemporal investigation of these swine M-segment variants revealed their circulation almost exclusively within North America between 2011 and 2016, i.e. they again appeared after the original assay had been published. However, among the 4,790 swine M-sequence submissions from North America from this period, the G247T variant represented only 3.9% (data not shown). It could not be suggested from the data whether the G247T strain was associated with certain outbreaks or just represented sporadically occurring minor variants in the background of the prevalent “standard” M-segment type.

Concerning the G247T M-segment submissions within the Avian data, the Korean H9N2 strains were the most striking. From the 150 informative H9N2 sequences available from Korea in the GISAID database within the 2003–2009 period, 47% held the G247T change (data not shown). Such a high prevalence might indicate a significant spatial cluster. Hence, from the viewpoint of assay inclusivity, H9N2 viruses with two M-segment strains circulated in Korean poultry, where the G247T H9N2 variant was potentially critical. Again, it could not be inferred from the submissions alone whether the H9N2 G247T strain really was co-dominant or merely represented a minor population in the background of the dominant G247 H9N2 viruses.

Despite these minor deviations, the data suggest that the re-assessed RT-qPCR assay is suitable as a method of choice for the generic detection of avian and swine IA viruses with an additional extended capacity for outbreak surveillance resulting from influenza A transmission to non-reservoir species including human zoonoses. However, albeit minor, the A88G and G247T (and G247A) mutations observed in Swine and Avian data warrant experimental evaluation. Unfortunately, our repository did not contain these rare IA virus strains. Therefore, the effect of the A88G and G247T/A variants on assay fitness remains to be investigated on real specimens. In any case, the M-segment positions 88 and 247 will be important targets for future *in silico* re-assessment.

Generally, proper oligonucleotide selection for microbial detection is a comprehensive procedure encompassing multiple steps and several software applications. Although various LS-MSA visualization, editing, and manipulation software applications are currently available, many of them require local installation and greater expertise, and sometimes command-line knowledge. Furthermore, derivative instruments for oligonucleotide evaluation, like quick LS-MSA filtering, variant composition assessment, and sequence sub-selection are generally sparse. Nor are these functions included in alignment viewers and editors. Therefore, performing these tasks is laborious and, for inexperienced users, they are even more complicated.

Our *in silico* re-assessment pipeline clearly illustrated the utility of the SequenceTracer in alignment sub-filtering with the ability to infer the important spatiotemporal characteristics of template variants with potentially adverse effects on a given diagnostic qPCR/RT-qPCR assay. The SequenceTracer enables a quick overview across the aligned sequences, stratification, compression, and personal sequence subset selection, mostly with a single mouse click. The algorithm is intuitive and requires no comprehensive expertise in bioinformatics. However, some minimal knowledge regarding LS-MSA preparation is necessary. The tool is available as a web application, it requires no installation and is platform independent.

The highly asymmetric distribution of the stratified data required the establishment of a threshold. A proper threshold level should be sufficiently low to also include minor, but significant, variants. However, it should be sufficiently high to exclude extremely rare variants or possible mistakes. Bearing in mind both of these extremes, we set the threshold level to ≥0.5% for each data pool. Therefore, we cannot exclude that such an empirical threshold adjustment might miss submissions with other important spatiotemporal characteristics like the aforementioned Korean H9N2 strains. However, the concatenated alignments are available as a Supplementary Dataset, so the reader can manage his own sequence tracing experiments.

The methodology presented relies to a large extent on multiple sequence alignments. Despite the obvious and generally accepted advances of LS-MSAs in diagnostic PCR/qPCR assay design, it should be kept in mind that they are burdened with multiple biases. One of the most important is a compositional bias leading to the unequal representation of the sequence variants^21^. The compositional bias is itself multicomponent and arises from various sources like: (i) purposeful bias towards antigenically^22^ and mutationally important strains; (ii) spatial and temporal biases, where outbreaks in certain geographic regions or epidemic seasons are sequenced more comprehensively than in others, and; (iii) redundancy where the same virus strain is re-sequenced or re-submitted. Another source of bias results from laboratory variants, i.e., recombination experiments, multiply-passaged strains, cell line-adapted variants, etc. or from sequencing artefacts of multiple characters, including low quality or ambiguous data, incorrect nucleotides, short artificial insertions or deletions, incorrect sequence directions, and short sequence stretches^14,23^. Although the majority of such low quality data can be easily identified and removed during alignment validation, some of the data originated from more hidden anomalies^24^. Despite these biases, the LS-MSA has a high corresponding value in the development and re-evaluation of diagnostic PCR/qPCR tests for the detection of highly variable viral templates. In addition, the methodology presented here clearly demonstrates the utility of the SequenceTracer in this process. The principles presented here should be considered a standard step in oligonucleotide quality assurance during assay design and re-assessment before routine use in a diagnostic laboratory.

## Methods

The re-assessment pipeline is summarised in the following steps (Fig. 1):

### Sequence Download

The IA virus M-segment sequences were downloaded from two databases GISAID’s (Global Initiative on Sharing All Influenza Data) EpiFlu^25^ and the Influenza Virus Database^26^ on 27 February 2018. The data were processed as five separate pools: (i) Human H3N2; (ii) Human pandemic H1N1 (H1N1pdm); (iii) Avian; (iv) Swine; and (v) Others (Table 1).

### Multiple Sequence Alignment

An LS-MSA was performed separately for each of the five data pools using the MAFFT version 7 online tool (Multiple Alignment with Fast Fourier Transform)^27,28^ by selecting the “Auto”, “Allow unusual symbols”, and “Adjust direction according to the first sequence” options. The largest FASTA files were aligned by the experimental version of the MAFFT 7. The aligned sequences were downloaded as.pir files.

### Alignment Processing

Alignment validation, trimming, and concatenation were performed with the AliView program^29^, and the data were saved as FASTA files. To evaluate the sequence variability of each of the five LS-MSAs, the sequences were trimmed to a 182 nucleotide-long stretch, which represents the entire amplicon of the SVIP-MP RT-qPCR assay^13^. Further, concatenated alignments containing only the forward primer-probe-reverse primer regions were prepared.

### Quantification of Nucleotide Variability

Sequence variability within the 182 nucleotide-long LS-MSAs was estimated separately for Human H3N2, Human H1N1pdm, Swine, Avian, and Others sequence pools according to the procedure reported previously^14^. Briefly, the positional nucleotide numerical summary (PNNS) and Entropy calculations were performed by using the PNNS and Entropy Calculator modules of the Alignment Explorer web application http://entropy.szu.cz:8080/EntropyCalcWeb/. The results of all five LS-MSAs were summarised in a combined column diagram (Fig. 2).

### Sequence Tracing and Inferring Assay Inclusivity

The particular concatenated forward primer-probe-reverse primer LS-MSAs were stratified by using the SequenceTracer module (and the Stratify function) of the Alignment Explorer http://entropy.szu.cz:8080/EntropyCalcWeb/. The data was graphically processed by using BioEdit v7.0.9.0^30^. The SequenceTracer source code is available at: https://gitlab.com/steve_michalik/entropycalc.

## Supplementary information

Sequence Tracer-Supplemetary information

Sequence Tracer-Supplemetary Dataset

## Data Availability

The concatenated Human H3N2, Human H1N1pdm, Avian, Swine and Others datasets generated and analysed during this study are provided in the Supplementary data of this article.
