# Supplementary material for: In silico re-assessment of a diagnostic RT-qPCR assay for universal detection of Influenza A viruses
Source: Sci Rep. 2019 Feb 7;9:1630. doi: 10.1038/s41598-018-37869-w (PMC6367508; doi:10.1038/s41598-018-37869-w)
Supplement: Supplementary file 1 — Sequence Tracer-Supplemetary information [file 41598_2018_37869_MOESM1_ESM.pdf]

In silico re-assessment of a diagnostic RT-qPCR assay for universal detection of Influenza A viruses

Alexander Nagy[1, 2,*], Tomáš Jiřinec[2], Helena Jiřincová[2], Lenka Černíková[1], Martina Havlíčková[2]

[1]State Veterinary Institute Prague, Czech Republic

[2]National Institute of Public Health, National Reference Laboratory for Influenza, Prague, Czech Republic

Supplementary information.

		SWINE 9,337							HUMAN H3N2 37,406							HUMAN H1N1pdm 16,948							AVIAN 21,530							Others 7,064						
	Pos. no.	A	G	C	U	GAP	SUM	Total	A	G	C	U	GAP	SUM	Total	A	G	C	U	GAP	SUM	Total	A	G	C	U	GAP	SUM	Total	A	G	C	U	GAP	SUM	Total
Forward	77	3	9293	0	0	41	9296	9337	8	36110	0	0	1286	36118	37404	3	16498	0	0	444	16501	16945	2	21083	1	4	440	21090	21530	1	6183	0	0	880	6184	7064
	78	0	9296	0	0	41	9296	9337	0	36121	0	0	1285	36121	37406	0	16504	0	1	441	16505	16946	2	21098	1	1	428	21102	21530	1	6183	0	0	880	6184	7064
	79	0	14	9282	0	41	9296	9337	0	5	36845	1	554	36851	37405	3	1	16504	0	439	16508	16947	1	3	21097	3	425	21104	21529	0	0	6775	0	289	6775	7064
	80	0	0	9060	235	41	9295	9336	0	0	36857	1	548	36858	37406	0	0	16498	10	435	16508	16943	1	0	21119	0	410	21120	21530	0	0	6777	2	285	6779	7064
	81	1	1	9294	0	41	9296	9337	0	0	36858	0	548	36858	37406	0	0	16512	0	435	16512	16947	2	0	21126	2	400	21130	21530	0	0	6779	0	285	6779	7064
	82	5	0	9291	0	41	9296	9337	0	0	36859	0	547	36859	37406	0	0	16512	0	435	16512	16947	0	4	21127	1	398	21132	21530	0	0	6778	1	285	6779	7064
	83	3	1	9291	1	41	9296	9337	0	0	36857	1	547	36858	37405	0	0	16510	1	435	16511	16946	4	3	21174	8	340	21189	21529	1	0	6777	1	285	6779	7064
	84	0	0	1	9295	41	9296	9337	0	0	1	36859	546	36860	37406	0	0	0	16518	429	16518	16947	3	1	5	21183	338	21192	21530	0	0	0	6780	284	6780	7064
	85	0	0	9294	2	41	9296	9337	1	0	36859	0	546	36860	37406	0	0	16524	0	423	16524	16947	1	4	21186	4	335	21195	21530	0	0	6779	1	284	6780	7064
	86	9300	0	0	0	37	9300	9337	36849	1	0	0	544	36850	37394	16527	0	0	0	419	16527	16946	21188	1	4	1	336	21194	21530	6778	1	0	1	284	6780	7064
	87	9291	0	7	2	37	9300	9337	36862	1	0	1	537	36864	37401	16532	1	0	0	414	16533	16947	21194	1	1	0	334	21196	21530	6786	0	0	0	278	6786	7064
	88	9230	69	0	1	37	9300	9337	36869	2	0	0	535	36871	37406	16525	8	0	0	414	16533	16947	21185	13	0	0	332	21198	21530	6781	2	1	2	278	6786	7064
	89	13	9290	0	0	34	9303	9337	0	36872	0	0	534	36872	37406	3	16541	0	3	400	16547	16947	7	21191	0	1	331	21199	21530	0	6787	0	0	277	6787	7064
	90	1	13	9289	0	34	9303	9337	0	1	36873	0	532	36874	37406	0	0	16549	0	398	16549	16947	2	3	21190	1	333	21196	21529	0	0	6791	0	273	6791	7064
	91	0	0	9298	5	34	9303	9337	2	0	36870	3	531	36875	37406	2	0	16546	1	397	16549	16946	2	4	21188	5	331	21199	21530	0	0	6789	4	271	6793	7064
	92	0	9301	2	0	33	9303	9336	2	36858	3	1	531	36864	37395	1	16549	0	0	396	16550	16946	3	21191	5	0	329	21199	21528	1	6783	10	0	269	6794	7063
	93	9304	0	0	0	33	9304	9337	36875	0	0	0	531	36875	37406	16550	1	0	0	396	16551	16947	21200	2	0	0	328	21202	21530	6794	2	0	0	268	6796	7064
Probe	227	0	9330	0	3	4	9333	9337	0	36927	0	1	478	36928	37406	0	16620	0	0	328	16620	16948	1	21324	1	0	203	21326	21529	0	6861	0	0	203	6861	7064
	228	0	3	0	9330	4	9333	9337	0	1	0	36927	478	36928	37406	0	0	0	16622	326	16622	16948	0	4	1	21322	202	21327	21529	0	1	0	6859	204	6860	7064
	229	0	9330	0	3	4	9333	9337	0	36927	0	1	478	36928	37406	0	16623	0	0	325	16623	16948	2	21323	0	2	202	21327	21529	2	6858	1	0	203	6861	7064
	230	0	1	9332	0	4	9333	9337	1	0	36927	0	478	36928	37406	0	1	16619	1	325	16621	16946	6	2	21315	2	203	21325	21528	0	1	6859	1	203	6861	7064
	231	0	0	9333	0	4	9333	9337	0	0	36928	0	478	36928	37406	0	0	16624	0	324	16624	16948	3	0	21318	4	203	21325	21528	0	0	6861	0	203	6861	7064
	232	6	4	9319	4	4	9333	9337	3017	0	33909	0	477	36926	37403	3	0	16609	12	324	16624	16948	12	3	21304	7	203	21326	21529	8	0	6839	14	203	6861	7064
	233	9332	1	0	0	4	9333	9337	36929	0	0	0	477	36929	37406	16623	1	0	0	324	16624	16948	21317	1	0	0	211	21318	21529	6860	1	0	0	203	6861	7064
	234	3	9330	0	0	4	9333	9337	1	36926	2	0	477	36929	37406	6	16617	0	0	325	16623	16948	26	21292	0	0	211	21318	21529	0	6860	1	0	203	6861	7064
Reverse	242	0	9335	0	0	2	9335	9337	2	36926	0	0	477	36928	37405	0	16623	0	0	324	16623	16947	5	21311	1	0	211	21317	21528	0	6858	0	0	206	6858	7064
	243	0	9335	0	0	2	9335	9337	0	36929	0	0	477	36929	37406	0	16624	0	0	324	16624	16948	0	21317	0	0	211	21317	21528	1	6857	0	0	206	6858	7064
	244	9326	9	0	0	2	9335	9337	36927	1	1	0	477	36929	37406	16619	5	0	0	324	16624	16948	21281	17	18	1	211	21317	21528	6856	2	0	0	206	6858	7064
	245	1	0	9330	4	2	9335	9337	0	0	36929	0	477	36929	37406	0	0	16624	0	324	16624	16948	2	1	21308	6	211	21317	21528	0	1	6857	0	206	6858	7064
	246	2	0	8	9325	2	9335	9337	0	0	23	36906	477	36929	37406	3	0	0	16621	324	16624	16948	9	2	1	21306	210	21318	21528	2	1	0	6855	206	6858	7064
	247	17	9111	2	203	2	9333	9335	1	36927	0	1	477	36929	37406	17	16604	0	3	324	16624	16948	118	21104	0	97	209	21319	21528	2	6851	0	5	206	6858	7064
	248	1	0	9334	0	2	9335	9337	0	0	36929	0	477	36929	37406	0	0	16624	0	324	16624	16948	0	0	21319	0	209	21319	21528	0	0	6856	2	206	6858	7064
	249	9334	1	0	0	2	9335	9337	36929	0	0	0	477	36929	37406	16619	0	0	0	329	16619	16948	21319	0	0	1	208	21320	21528	6856	1	1	1	205	6859	7064
	250	76	9257	0	1	2	9334	9336	81	36848	0	0	477	36929	37406	11	16606	0	0	330	16617	16947	202	21116	0	2	208	21320	21528	39	6818	0	1	205	6858	7063
	251	0	0	9335	0	2	9335	9337	0	0	36928	0	477	36928	37405	0	0	16618	0	330	16618	16948	4	1	21314	2	207	21321	21528	0	0	6858	1	205	6859	7064
	252	0	9315	0	20	2	9335	9337	0	36928	0	1	477	36929	37406	0	16618	0	0	330	16618	16948	0	21321	0	0	206	21321	21527	2	6855	1	1	205	6859	7064
	253	0	0	2	9333	2	9335	9337	0	0	2	36926	477	36928	37405	0	1	28	16588	330	16617	16947	1	2	24	21292	209	21319	21528	0	0	2	6712	350	6714	7064
	254	9335	0	0	0	2	9335	9337	36928	0	1	0	477	36929	37406	16617	0	0	0	330	16617	16947	21315	0	4	0	209	21319	21528	6714	0	0	0	350	6714	7064
	255	5	9326	2	1	2	9334	9336	4	36917	0	5	477	36926	37403	0	16616	0	0	330	16616	16946	16	21291	3	7	209	21317	21526	2	6702	3	7	350	6714	7064
	256	9322	13	0	0	2	9335	9337	36924	5	0	0	477	36929	37406	16610	8	0	0	330	16618	16948	21273	43	0	4	208	21320	21528	6713	1	0	0	350	6714	7064
	257	1	0	9334	0	2	9335	9337	1	0	36928	0	477	36929	37406	0	0	16618	0	330	16618	16948	11	1	21309	0	207	21321	21528	0	0	6714	0	350	6714	7064
	258	1	9331	1	2	2	9335	9337	0	36929	0	0	477	36929	37406	1	16614	3	0	330	16618	16948	5	21311	0	2	206	21318	21524	0	6712	1	1	350	6714	7064

Figure S1. PNNS for the primers and the probe sequences in five data pools. Positions with elevated variations are bordered in red.

S2a

Human H1N1pdm 16,948

Group Number	Variant Count	Frequency %	80 90 230 250<br>...|....|....|... ...|.... ...|....|....|...
			GGCCCCCTCAAAGCCGA---GTGCCCAG---GGACTGCARCGTAGACG
1	16344	96,436	.................~~~........~~~.................
2	28	0,165	.................~~~........~~~...........c.....
3	17	0,100	.................~~~........~~~.....a...........
4	12	0,071	.................~~~.....t..~~~.................
5	11	0,065	.................~~~........~~~.................
6	10	0,059	...t.............~~~........~~~.................
7	8	0,047	...........g.....~~~........~~~.................
8	8	0,047	.................~~~........~~~..............g..
9	6	0,035	.................~~~.......a~~~.................
10	5	0,030	.................~~~........~~~..g..............
11	3	0,018	.................~~~.....a..~~~.................
12	3	0,018	............a....~~~........~~~.................
13	3	0,018	.................~~~........~~~.....t...........
14	3	0,018	a................~~~........~~~.................
15	3	0,018	.................~~~........~~~....a............
16	3	0,018	.................~~~........~~~................c
17	3	0,018	..a.........t....~~~........~~~.................
18	2	0,012	..............a..~~~........~~~.................
19	1	0,006	.................~~~...g....~~~.................
20	1	0,006	..........g......~~~........~~~.................
21	1	0,006	.................~~~...t....~~~.................
22	1	0,006	.................~~~......g.~~~.................
23	1	0,006	......t..........~~~........~~~.................
24	1	0,006	................g~~~........~~~.................
25	1	0,006	..............t..~~~........~~~.................
26	1	0,006	.................~~~........~~~...........g.....
27	1	0,006	...............a.~~~........~~~.................
28	1	0,006	.t...............~~~........~~~.................
29	1	0,006	..g..............~~~........~~~.................
30	1	0,006	.................~~~........~~~................a
outgroup1	17	0,100	
outgroup2	125	0,738	
excluded	322	1,900	

S2b

Swine 9,337

Group Number	Variant Count	Frequency %	80 90 230 250<br>...|....|....|... ...|.... ...|....|....|...
			GGCCCCCTCAAAGCCGA---GTGCCCAG---GGACTGCARCGTAGACG
1	8560	91,678	.................~~~........~~~.................
2	235	2,517	...t.............~~~........~~~.................
3	202	2,163	.................~~~........~~~.....t...........
4	73	0,782	.................~~~........~~~.................
5	69	0,739	...........g.....~~~........~~~.................
6	19	0,203	.................~~~........~~~..........t......
7	17	0,182	.................~~~........~~~.....a...........
8	12	0,129	..g..............~~~........~~~.................
9	12	0,129	.................~~~........~~~..............g..
10	9	0,096	.................~~~........~~~..g..............
11	8	0,086	.................~~~........~~~....c............
12	6	0,064	.................~~~.....a..~~~.................
13	4	0,043	.................~~~.....g..~~~.................
14	4	0,043	.................~~~........~~~...t.............
15	4	0,043	............a....~~~........~~~.................
16	4	0,043	.................~~~........~~~.............a...
17	3	0,032	.................~~~.......a~~~.................
18	3	0,032	......a..........~~~........~~~.................
19	3	0,032	.................~~~tgt.....~~~.................
20	3	0,032	.....a.......gt..~~~........~~~.................
21	2	0,021	.................~~~........~~~................t
22	2	0,021	.................~~~........~~~...........c.....
23	2	0,021	.................~~~........~~~.............c...
24	2	0,021	.................~~~.....t..~~~.................
25	2	0,021	..........c.ag...~~~........~~~.................
26	2	0,021	.................~~~........~~~.....c...........
27	1	0,011	.................~~~...g....~~~.................
28	1	0,011	.....a...........~~~........~~~.................
29	1	0,011	.............a...~~~......g.~~~.................
30	1	0,011	.................~~~........~~~.............t...
31	1	0,011	.................~~~........~~~................a
32	1	0,011	...............c.~~~........~~~.................
33	1	0,011	a................~~~........~~~.................
34	1	0,011	.................~~~.....t..~~~.....t...........
35	1	0,011	.................~~~........~~~....a............
36	1	0,011	a.g..............~~~........~~~.................
37	1	0,011	.......c.........~~~........~~~.................
38	1	0,011	....g............~~~........~~~.................
39	1	0,011	....a............~~~........~~~.................
40	1	0,011	.................~~~........~~~...............a.
41	1	0,011	.................~~~........~~~...a.............
42	1	0,011	a.............t..~~~........~~~.................
43	1	0,011	..........tt.....~~~........~~~.................
44	1	0,011	.....a.......gt..~~~........~~~.............a...
45	1	0,011	.................~~~........~~~......a.t........
46	1	0,011	........t......c.~~~........~~~.................
47	1	0,011	......t..........~~~........~~~.................
48	1	0,011	......g..........~~~........~~~.................
49	1	0,011	..........t......~~~........~~~.................
50	1	0,011	..........c.ag...~~~........~~~.................
51	1	0,011	.................~~~........~~~................c
52	1	0,011	..g..............~~~........~~~.................
53	1	0,011	........t........~~~........~~~.................
54	1	0,011	.................~~~........~~~.......g.........
outgroup1	6	0,064	
outgroup2	39	0,418	
excluded	2	0,021	

S2c

AVIAN 21,530			
Group Number	Variant Count	Frequency %	80 90 230 250<br>...|....|....|... ...|.... ...|....|....|...
			GGCCCCCTCAAAGCCGA---GTGCCCAG---GGACTGCARCGTAGACG
1	20356	94,547	.................~~~.......~~~..................
2	185	0,859	.................~~~.......~~~..................
3	113	0,525	.................~~~.......~~~......a...........
4	96	0,446	.................~~~.......~~~......t...........
5	34	0,158	.................~~~.......~~~...............g..
6	21	0,098	.................~~~.......~~~a.................
7	20	0,093	.................~~~.......~~~............c.....
8	17	0,079	.................~~~.......~~~...g..............
9	13	0,060	...........g.....~~~.......~~~..................
10	11	0,051	.................~~~.......~~~...c..............
11	11	0,051	.................~~~.......~~~................a.
12	9	0,042	.................~~~.......~~~..............a...
13	9	0,042	.................~~~.......~~~.....a............
14	9	0,042	.................~~~.......~~~...............g..
15	8	0,037	.................~~~.....a.~~~..................
16	7	0,033	.................~~~.....t.~~~..................
17	6	0,028	......t..........~~~.......~~~..................
18	6	0,028	.................~~~...a...~~~..................
19	5	0,023	.................~~~.......~~~....t.............
20	4	0,019	.................~~~....t..~~~..................
21	4	0,019	...............c.~~~.......~~~..................
22	4	0,019	.................~~~.......~~~.a................
23	4	0,019	.................~~~.......~~~a.....a...........
24	4	0,019	.................~~~.......~~~..............t...
25	4	0,019	.......c.........~~~.......~~~..................
26	4	0,019	t................~~~.......~~~..................
27	3	0,014	......a..........~~~.......~~~..................
28	3	0,014	............a....~~~.......~~~..................
29	3	0,014	..g..............~~~.......~~~..................
30	3	0,014	.................~~~.....a.~~~............c.....
31	3	0,014	.................~~~....a..~~~..................
32	2	0,009	........t........~~~.......~~~..................
33	2	0,009	..............t..~~~.......~~~..................
34	2	0,009	.................~~~.......~~~..........t.......
35	2	0,009	.................~~~...g...~~~..................
36	2	0,009	a................~~~.......~~~..................
37	2	0,009	.................~~~..a....~~~..................
38	2	0,009	.........c..ag...~~~.......~~~..................
39	2	0,009	......g..........~~~.......~~~..................
40	2	0,009	.......a.........~~~.......~~~..................
41	2	0,009	.................~~~.......~~~..............a..a
42	2	0,009	.................~~~.......~~~............g.....
43	2	0,009	.................~~~.......~~~..............c...
44	2	0,009	.................~~~.......~~~...c......a.......
45	2	0,009	.....g..g.....g..~~~.....g.~~~...c.........c.t..
46	2	0,009	.................~~~...t...~~~..................
47	1	0,005	.......a.........~~~.......~~~..................
48	1	0,005	..t....ct........~~~.......~~~..................
49	1	0,005	....t............~~~.......~~~..................
50	1	0,005	..a.t............~~~.......~~~..................
51	1	0,005	.................~~~.......~~~.................t
52	1	0,005	..t...gg.........~~~.......~~~..................
53	1	0,005	.................~~~a......~~~..................
54	1	0,005	.................~~~..t....~~~...ca.....a.......
55	1	0,005	..........g...tc.~~~.......~~~..................
56	1	0,005	..............a..~~~.......~~~..................
57	1	0,005	........g........~~~.......~~~.................a
58	1	0,005	c..............a.~~~.......~~~..................
59	1	0,005	...............ag~~~.......~~~..................
60	1	0,005	................g~~~.......~~~..................
61	1	0,005	.t...............~~~.......~~~..................
62	1	0,005	.................~~~.......~~~...............t..
63	1	0,005	......t..gc......~~~.......~~~..................
64	1	0,005	.................~~~.......~~~........t.........
65	1	0,005	.............a...~~~.......~~~..................
66	1	0,005	....a............~~~.......~~~..................
67	1	0,005	.a...............~~~.......~~~..................
68	1	0,005	............ag...~~~.......~~~..................
69	1	0,005	.................~~~c......~~~..................
70	1	0,005	.................~~~.......~~~....g.............
71	1	0,005	.................~~~..t....~~~.....g............
72	1	0,005	.................~~~.......~~~.c................
73	1	0,005	.................~~~.......~~~..........g.......
74	1	0,005	.................~~~.......~~~......t.......a...
75	1	0,005	..............g..~~~.......~~~..................
76	1	0,005	......t......a...~~~.......~~~..................
77	1	0,005	............t....~~~.......~~~..................
78	1	0,005	.................~~~.....a.~~~............c.....
79	1	0,005	.................~~~.......~~~............a.....
80	1	0,005	....a.........a..~~~.......~~~..................
81	1	0,005	..t..............~~~.......~~~..................
82	1	0,005	.................~~~.......~~~...c..a...........
83	1	0,005	........a........~~~.......~~~..................
84	1	0,005	.................~~~.......~~~.....g............
85	1	0,005	.................~~~.......~~~..............t..a
86	1	0,005	.c...............~~~.......~~~..................
87	1	0,005	..............t..~~~.......~~~................g.
88	1	0,005	.................~~~.......~~~.................a
89	1	0,005	.................~~~.......~~~.....c............
90	1	0,005	.................~~~.......~~~...t..............
91	1	0,005	...a.............~~~.......~~~..................
92	1	0,005	.................~~~.......~~~..........a.......
93	1	0,005	.....ga..........~~~.......~~~..................
94	1	0,005	.................~~~.c.....~~~..................
95	1	0,005	.................~~~.g.....~~~..................
96	1	0,005	.................~~~.......~~~.a...........c....
97	1	0,005	.....t...........~~~.......~~~..................
98	1	0,005	.............t...~~~.......~~~..................
99	1	0,005	.................~~~.......~~~..............t..t
100	1	0,005	.....g..g.....ga.~~~.....g.~~~...c.........c.t..
outgroup1	15	0,070	
outgroup2	267	1,240	
excluded	185	0,859	

S2d

Others 7,064			
Group Number	Variant Count	Frequency %	80 90 230 250<br>...|....|....|... ...|.... ...|....|....|...
			GGCCCCCTCAAAGCCGA---GTGCCCAG---GGACTGCARCGTAGACG
1	5924	83,862	.................~~~........~~~.................
2	34	0,481	.................~~~........~~~.................
3	11	0,156	.................~~~.....t..~~~.................
4	8	0,113	.................~~~.....a..~~~.................
5	8	0,113	...............c.~~~........~~~.................
6	6	0,085	.................~~~........~~~.............t...
7	5	0,071	.................~~~........~~~.....t...........
8	2	0,028	.................~~~........~~~.............c...
9	2	0,028	.................~~~........~~~...........c.....
10	2	0,028	.................~~~..a.....~~~.................
11	2	0,028	.................~~~........~~~.............a...
12	2	0,028	...........g.....~~~........~~~.................
13	2	0,028	.................~~~........~~~..........a......
14	2	0,028	.................~~~........~~~.....a...........
15	2	0,028	...t.............~~~........~~~.................
16	1	0,014	.................~~~........~~~.......t.........
17	1	0,014	...........t.....~~~........~~~.................
18	1	0,014	.................~~~........~~~..............g..
19	1	0,014	.................~~~.......c~~~........t....c..t
20	1	0,014	...........t.....~~~.gc.....~~~.........t.......
21	1	0,014	.................~~~........~~~................c
22	1	0,014	..............t.g~~~........~~~.................
23	1	0,014	.................~~~......g.~~~.................
24	1	0,014	.................~~~........~~~.......c.........
25	1	0,014	......t..........~~~........~~~.................
26	1	0,014	..............t..~~~........~~~.................
27	1	0,014	......a..........~~~........~~~.................
28	1	0,014	.................~~~...t....~~~.................
29	1	0,014	...........c.....~~~........~~~.................
30	1	0,014	a................~~~........~~~.................
31	1	0,014	.........t.......~~~........~~~.................
32	1	0,014	.................~~~........~~~....a............
33	1	0,014	.................~~~........~~~..........c......
34	1	0,014	.................~~~........~~~..........t......
outgroup1	2	0,028	
outgroup2	839	11,877	
excluded	192	2,718	

Figure S2. Alignment stratification. The concatenated MSAs of Human H1N1pdm (**S2a**), Swine (**S2b**), Avian (**S2c**), and Others (**Sd**) were decomposed into individual sequence variants by using the "Stratify" function of the SequenceTracer. The figures show the number of M-segment groups and the frequency of each variant in descending order along with a representative sequence of each group aligned to the primers and probe-binding regions (5'→3'). Note that the original probe is in reverse direction, i. e. CTGGGCAC. The alignment was prepared in the Graphic View mode of the BioEdit program. For clarity, the primers and probe sequences were separated by tildes and numbered according their M-segment positions. The dots indicate an identical nucleotide. The bold grey bar designates the threshold (≥0.5%). "Outgroup 1" contains sequences with at least one uncertainty (R, Y, N, etc), and "outgroup 2" encompasses an incomplete database of submissions. The "excluded" submissions contain no data.
